# Quinolinic acid selectively induces apoptosis of human astrocytes: potential role in AIDS dementia complex

**DOI:** 10.1186/1742-2094-2-16

**Published:** 2005-07-26

**Authors:** Gilles J Guillemin, Lily Wang, Bruce J Brew

**Affiliations:** 1 Centre for Immunology, St Vincent's Hospital, Sydney, Australia; 2 Department of Neurology, St Vincent's Hospital, Sydney, Australia; 3 University of New South Wales, Faculty of Medicine, Sydney, Australia

**Keywords:** Human, astrocyte, apoptosis, quinolinic acid, caspase 3, AIDS dementia complex

## Abstract

There is evidence that the kynurenine pathway (KP) and particularly one of its end products, quinolinic acid (QUIN) play a role in the pathogenesis of several major neuroinflammatory diseases, and more particularly AIDS dementia complex (ADC). We hypothesized that QUIN may be involved in astrocyte apoptosis because: 1) apoptotic astrocytes have been observed in the brains of ADC patients, 2) ADC patients have elevated cerebrospinal fluid QUIN concentrations, and 3) QUIN can induce astrocyte death. Primary cultures of human fetal astrocytes were treated with three pathophysiological concentrations of QUIN. Numeration of apoptotic cells was assessed using double immunocytochemistry for expression of active caspase 3 and for nucleus condensation. We found that treatment of human astrocytes with QUIN induced morphological (cell body shrinking) and biochemical changes (nucleus condensation and over-expression of active caspase 3) of apoptosis. After 24 hours of treatment with QUIN 500 nM and 1200 nM respectively 10 and 14% of astrocytes were undergoing apoptosis. This would be expected to lead to a relative lack of trophic support factors with consequent neuronal dysfunction and possibly death. Astroglial apoptosis induced by QUIN provides another potential mechanism for the neurotoxicity of QUIN during ADC.

## Findings

The kynurenine pathway (KP) is a major route of L-tryptophan catabolism, resulting in the production of nicotinamide adenine dinucleotide and other neuroactive intermediates [1]. Of the metabolites, the N-methyl-D-aspartate (NMDA) receptor agonist and neurotoxin quinolinic acid (QUIN) is likely to be one of the most important. There is evidence that QUIN is involved in the neurocytotoxicity associated with several major inflammatory brain diseases [2,3] such as AIDS dementia complex (ADC) [4,5] and other viral brain infections [2]. In the central nervous system (CNS), astrocytes play essential roles including metabolic homeostasis especially providing neurotrophic support, detoxification, maintenance of the blood brain barrier and immune response. During the brain inflammation associated with ADC, many mediators are released and astrocytes are activated leading to their cellular hypertrophy and/or proliferation [6]. For some astrocytes, this prolonged activation may induce apoptosis and the death of these reactive astrocytes can directly and/or indirectly affect functions and survival of the neighbouring neurons and astrocytes [7]. The consequences of astrocyte apoptosis could be either neuroprotective [8] or neurodamaging [7,9]. Apoptosis of astrocytes has been described in the brains of patients with ADC [10-12]. Furthermore, in ADC both brain parenchyma and cerebrospinal fluid (CSF) concentrations of QUIN are strongly elevated [4,5,13] respectively 300 and 100 fold compared to controls. The HIV-1 proteins Nef and Tat induce macrophages to produce QUIN [14]. The association between brain cell apoptosis and increased levels of QUIN have been found in various other neurodegenerative diseases. We therefore hypothesized that QUIN could be linked with astrocyte apoptosis.

**Figure 1:**
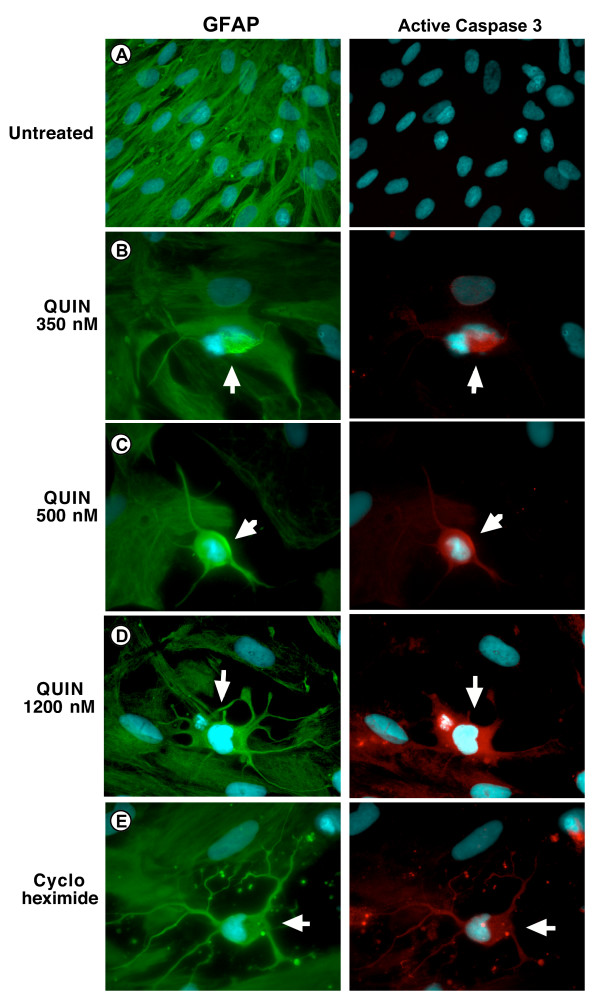
Detection of apoptotic astrocytes using immunocytochemistry. Double staining for GFAP (Left column/green)) and active caspase 3 (Right column/red). Nucleuses are stained in blue with DAPI. **A) **Untreated cells; **B) **treated with QUIN 350 nM; **C) **treated with QUIN 500 nM; **D) **treated with QUIN 1200 nM; **E) **treated with cycloheximide 20 μg/ml for 24 hours. Arrows point apoptotic astrocytes.

**Figure 2:**
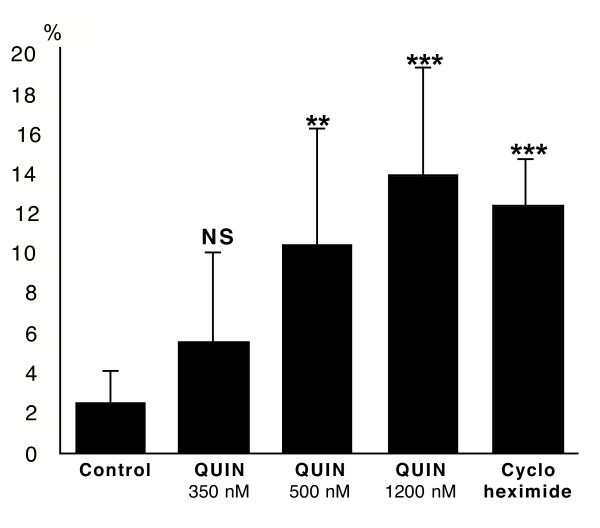
Numeration of apoptotic astrocytes using immunocytochemistry. Histogram showing the percentage of apoptotic astrocytes relative to the total number of astrocytes after 24 hours of treatment. Mean values and standard errors were calculated for each treatment. Unpaired *t *tests were used to analyse the significance of differences between pairs of the three treatments. A *p *value of <0.05 was regarded as statistically significant. *p *value between controls and QUIN 350 nM, QUIN 500 nM, 1200 nM treated slides were respectively 0.07 (NS), 0.04 (**) and 0.004 (***); and 0.002 (***) between controls and cycloheximide treated slides. *p *values between QUIN 500 nM, 1200 nM and cycloheximide treated slides were not significantly different.

We used primary cultures of human fetal astrocytes treated with three pathophysiological concentrations of QUIN respectively 350, 500 and 1200 nM (similar to those in brain parenchyma of ADC patients [15]) and assessed them for apoptosis with immunocytochemistry. We found that 99% of the cells were GFAP positive (green staining, Fig. 1, left column) demonstrating the high purity of the primary cultures of human astrocytes. No staining was detected for CD68, MAP2, factor VIII or 5B5 (data not included). Apoptotic cells started to be detected after only 6 hours(data not presented). Apoptotic astrocytes displayed an atypical morphology with a shrunken body and abnormal processes. Moreover, most of these cells are in the process of detaching from the culture flask. These apoptotic cells were easily spotted due to their condensed and very bright nucleus with the DAPI staining (cyan staining, Fig. 1). All these cells also displayed a strong cytoplasmic and perinuclear staining for the anti-active caspase-3 (red staining, Fig. 1, right column). The percentage of apoptotic cells was calculated after 24 hours of treatment (as described in the methodological section below) (Fig. 2). After 24 hours, the percentage of apoptotic cells in cycloheximide-treated slides was increased 5-fold whilst in QUIN 350, QUIN 500 and QUIN 1200 nM-treated slides they were respectively increased by 2.2, 4.2 and 5.6-fold compared to baseline. The *p*-values between the cycloheximide-treated slides and the QUIN 500 and 1200 nM-treated slides were not significant 24 hours but were significant between control slides and treated slides, with exception for QUIN 350 nM (see Fig. 2 legend).

This study demonstrated that treatments with QUIN in pathophysiological concentrations induced morphological and biochemical changes of apoptosis in a sub-group of human astrocytes in a dose dependent manner. Some issues can be raised in term of potential experimental limitations of our *in vitro *results. Firstly, there are two known subtypes of astrocytes [16] but only type1 astrocytes can be grown in the primary cultures obtained by our method [17]. Differences between these subtypes are not well known and it is possible that they may have a different sensitivity to QUIN. Secondly, it can be argued that fetal and adult astrocytes may have a different sensitivity to QUIN. This is unlikely because we previously showed the high degree of similarity between immature and mature astrocytes [18,19]. Moreover, in ADC brains high levels of QUIN [15] are associated with a high rate of apoptosis of adult astrocytes [10]. Finally, the limited number of apoptotic astrocytes (<15%) implies that only a subset of astrocytes is susceptible to QUIN toxicity. The receptors and signalling pathways that initiate astroglial apoptosis has not been identify yet. However, two main mechanisms may potentially be involved. The first is the activation of NMDA receptors by QUIN [4], which is already known to mediate neuronal apoptosis with caspase-3 activation [20-23]. However, the presence of NMDA receptors on astrocytes is very controversial [24,25]. A similar pathway may be involved in neurons and astrocytes, but the presence of functional NMDA receptors on astrocytes still has to be proved. The second possibility, which represent another important aspect of QUIN toxicity, is lipid peroxidation [26]. QUIN in concentration as low as 120 nM induces lipid peroxidation and formation of free radicals leading to neuronal death [27]. This second mechanism is more likely to be involved astrocyte apoptosis induced by QUIN.

Interestingly, our *in vitro *results on QUIN toxicity in human astrocytes correlate well with previous *in vivo *studies using animal models. After QUIN injection into the rat brain, a reduction in the density of GFAP-immunoreactivity of astrocytes occurs early (within 6 hours of injection) suggesting astrocytic death; which could be associated with necrosis rather than apoptosis [28,29]. Another *in vivo *study using fetuses derived from ewes and damaged by hypoxia and hypoglycemia showed a significant increase in QUIN concentration associated with reduction in GFAP in fetal brain [30]. However, whether QUIN is able to induce astrogliosis and/or astrocytosis is still controversial [28,29]. QUIN can increase local inflammation by inducing production of cytokines and chemokines by human astrocytes [18]. QUIN increases neuronal release of glutamate and inhibits its uptake by astrocytes, leading to an excessive glutamate concentration in the neuronal microenvironment and secondly to neurotoxicity [9].

It is still debated whether astroglial activation is beneficial or detrimental to neurons [7]. For some astrocytes, this activation will lead to an early cellular death, as we showed here with QUIN. This may be part of a selective neuroprotective mechanism because on one hand reactive astrocytes may contribute to a decline of neurological function through various mechanisms [8]. On the other hand, the loss of astrocytes compromises their beneficial effects on neuronal function and survival [31]. The results of this study are biologically relevant because brain cell apoptosis (including neurons and astrocytes) and increased levels of QUIN have been found associated in various neurodegenerative diseases and brain disorders such as ADC [10], Alzheimer's disease [32,33], cerebral malaria [34], and traumatic brain injury [35].

A large majority of the *in vivo *or *ex vivo *studies concerning astrocyte apoptosis are related to HIV brain infection and ADC [12]. Thompson *et al*. [10] found that there is a correlation between an increased number of HIV DNA-positive astrocytes and an increased number of apoptotic astrocyte and rapid progression of patients to dementia. Furthermore, QUIN production is directly related to the viral load in patients with ADC [36]. There is strong evidence that ADC is associated with NMDA receptor activation [37,38]. We previously demonstrated that HIV-1 proteins Nef and Tat lead to production of high levels of QUIN by human macrophages [14]; that inhibition of QUIN production by HIV-1 infected macrophages strongly reduces neurotoxicity [39]; and finally that QUIN can amplify neuroinflammation and increase astroglial expression of HIV-1 co-receptors [18]. Interestingly, viral induction of IDO in human macrophages differs according to particular HIV-1 isolates [40] and similarly diverse HIV-1 primary isolates have different effects on astrocyte apoptosis [11]. The present study provides a direct link between the astrocyte apoptosis and high levels of QUIN in ADC brains. These data provide a new mechanism by which HIV-1 may be involved in astrocyte apoptosis directly and indirectly via QUIN production. The characterisation of molecular pathways leading to QUIN-induced astrocyte apoptosis has the potential for developing agents that reduce glial and neuronal death related to QUIN-toxicity. Development of pharmacological agents targeting specific KP enzymes was reviewed recently [41].

All cell culture media and additives were from Invitrogen (Melbourne, VIC, Australia) unless otherwise stated. QUIN, DAPI, cycloheximide was obtained from Sigma-Aldrich Chemical Co. (Sydney, NSW, Australia). anti-active caspase-3 antibody (polyclonal) was from BD Pharmingen. Mouse mAb anti-GFAP (clone GA-5) was obtained from Novacostra (Newcastle, UK). Secondary goat anti-mouse IgG and anti-rabbit Alexa 488 (green) or Alexa 594 (red) conjugated antibodies were purchased from Molecular Probes (Eugene, OR, USA). All commercial antibodies were used at the concentrations recommended by the manufacturer. Human fetal brains were obtained from 16 to 19 week old fetuses collected following informed consent. Astrocytes were prepared using a protocol adapted from previously described methods [17]. The experiments were carried out in triplicate throughout. Initially, cells were incubated in serum-free AIM-V. The negative control cells were incubated in AIM-V only. The QUIN group was incubated in 350, 500 and 1200 nM QUIN in AIM-V. The positive control cells were incubated in cycloheximide (20 μg/ml) [42] in AIM-V. Dose-response and time course (3, 6, 12, 24, 48, 72 hours) have been done for QUIN and cycloheximide (data not shown). The optimal time of incubation was 24 hours for the active caspase 3 detection. QUIN concentrations between 350 nM to 1200 nM are known to be found in patients with ADC [15]. The characterization of human brain cell using immunocytochemistry was previously described [17]. The following three controls were performed for each labelling experiment: 1) isotypic antibody controls, 2) incubation with only the secondary labelled antibodies, and 3) estimation of auto-fluorescence of unlabelled cells. The cell counting was performed in a blinded manner. The whole controls and treated chamber slides were counted. Enumeration for each slide was rechecked by the experimenter and cells classified according to the following scheme: DAPI staining for total cell number, GFAP immunoreactivity for astrocytes, and active caspase 3 immunoreactivity together with DAPI for apoptotic cells. Experiments were done in triplicates using brain cells from three different brain tissues. Mean values and standard errors were calculated for each treatment and the results were plotted on a histogram (Fig. 2). Unpaired *t *tests were performed on the results obtained at 24 hours. Student's t-test was used to analyse the significance of differences between pairs of the three treatments. A *p *value of <0.05 was regarded as statistically significant.

## List of abbreviations

AIDS dementia complex (ADC), cerebrospinal fluid (CSF), 4',6-diamidino-2-phenylindole (DAPI), Glial fibrillary acid protein (GFAP), human immunodeficiency virus (HIV), kynurenine pathway (KP), NMDA (N-methyl-D-aspartate), Quinolinic acid (QUIN).

## Competing interests

The author(s) declare that they have no competing interests.

## Authors' contributions

GG was responsible for conception and planning of the experiments, for performing the immunocytochemical study and for writing of the manuscript. LW was growing the primary cultures of human astrocytes and did the various treatments. BJB contributed to the interpretation of the results, discussion and writing of the manuscript.
